# Successful treatment by adding thalidomide to meglumine antimoniate in a case of refractory anthroponotic mucocutaneous leishmaniasis

**DOI:** 10.1016/j.ijpddr.2019.08.007

**Published:** 2019-08-27

**Authors:** Vahid Mashayekhi Goyonlo, Sadegh Vahabi-Amlashi, Faezeh Taghavi

**Affiliations:** a Department of Dermatology, Faculty of Medicine, Mashhad University of Medical Sciences, Mashhad, Iran; b Cutaneous Leishmaniasis Research Center, Mashhad University of Medical Sciences, Mashhad, Iran

**Keywords:** Mucocutaneous Leishmaniasis, Leishmania tropica, Thalidomide

## Abstract

Mucosal leishmaniasis (ML) is mostly associated with Leishmania braziliensis; however, a few cases of Leishmania tropica induced mucocutaneous leishmaniasis have been reported. The standard treatment for leishmaniasis is pentavalent antimonials, but several other drugs for resistant cases have been proposed including amphotericin and miltefosine. Here we present a case of multiple treatment resistant mucocutaneous leishmaniasis with nasal involvement caused by L. tropica; cure was not achieved by multiple treatments and was eventually improved by adding thalidomide to Meglumine Antimoniate (Glucantime). To the best of our knowledge use of thalidomide in humans for leishmaniasis treatment is reported here for the first time.

## 1 Summary

Most common causes of leishmaniasis in Iran are Leishmania major and Leishmania tropica that usually cause cutaneous leishmaniasis, however a few cases of L. tropica induced mucocutaneous leishmaniasis with oro-mucosal lesions have been reported that was treated by intravenous infusion of amphotericin B. Here we present a case of refractory mucocutaneous leishmaniasis with nasal involvement caused by *L. tropica*, which was eventually improved with thalidomide.

## 2 Case presentation

**Fig. 1:**
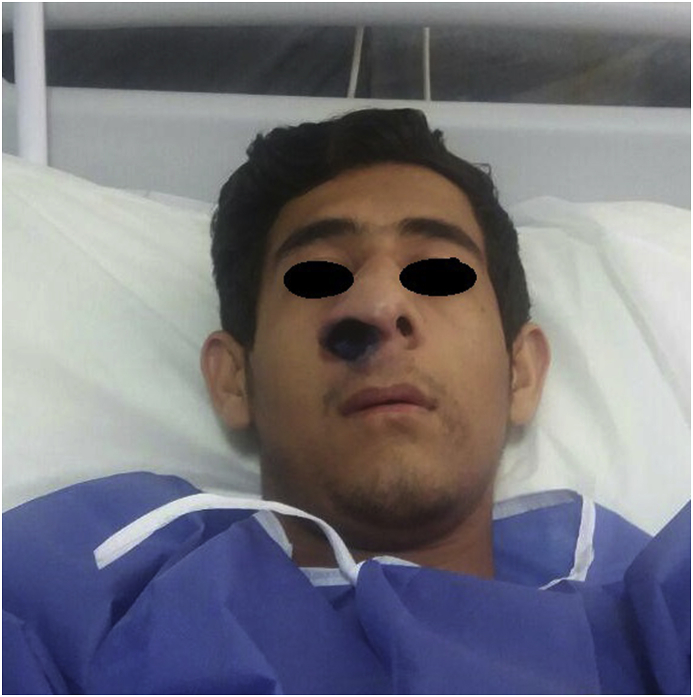
An indurated mass with overlying crust in the right nasal nare.

A 20-year-old man, accounting student, presented to cutaneous leishmaniasis clinic in Imam-Reza hospital Mashhad, Iran with an edematous mass in the right nasal nare. The patient had a history of cutaneous leishmaniasis 9 years ago (2009) on his chin and left forearm that was improved as a local outpatient treatment (with intralesional injection of meglumine antimoniate). However, after a short time, indurated mass on his left nasal nare developed. The new lesion showed leishmania parasite in the direct smear and was treated with one course of intramuscular meglumine antimoniate with remission of this lesion. Nevertheless, after one month, an indurated right nasal nare mass plus dyspnea and nocturnal snoring appeared. Nasal endoscopy sampling confirmed mucosal involvement by leishmaniasis. This bothersome mucocutaneous lesion (Fig. 1) did not cure over the last few years in spite of size reduction and temporary remission during various treatments, including a few course of intramuscular meglumine antimoniate (glucantime), amphotericin B deoxycholate and amphotericin B liposomal.

His Immune competency laboratory test included the nitroblue tetrazolium (NBT), the dihydrorhodamine (DHR) test, blood flow cytometry T cell subtype and serum hemolytic complement (CH50) activity were within normal limit except serum Immunoglobulin E, which was more than 500. Direct smears of the lesions were repeatedly positive and the polymerase chain reaction (PCR) reported the responsible parasite to be *L. tropica*. He also had anti-leishmania antibodies in his serum, but abdominal Ultrasound and bone marrow aspiration were negative and ruled out visceral leishmaniasis. Considering the location of the lesion, sinuses and nasal coronal CT scans reported no pathologies.

In 2016, he underwent surgical excision of the lesion and received oral miltefosine 150 mg daily for two months but the lesion did not resolve completely and recurred after 3 month.

**Fig. 2:**
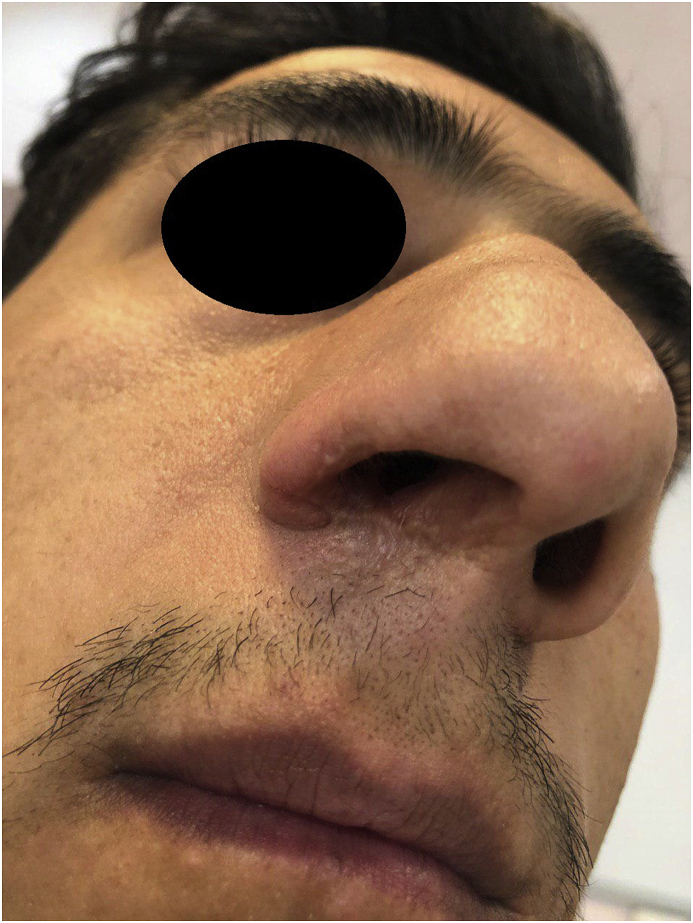
Residual scar in the right nasal nare after treatment.

Finally, in March 2018 the patient was readmitted to the dermatology department of *Imam-Reza* hospital with the diagnosis of multiple treatment resistant, *L. tropica* induced mucocutaneous leishmaniasis.At the time of admission, the patient weighed 66 kg and the presence of Leishman bodies was confirmed in the direct smear of the lesion. He was treated with combination of intramuscular glucantime (850 mg daily for 28 days) and oral thalidomide (100 mg daily for two months).Given that the first experience of this combination therapy, the minimum therapeutic dose of these drugs was used. During the first month of treatment the size of the lesion decreased significantly and during the second month the induration of the lesion disappeared and the atrophic and some retractive scar remained.No adverse effects observed during this combination therapy and the direct smears from the lesion were negative after discharge and at third and sixth months after the beginning of the treatment. No signs or symptoms of recurrence until now (February 2019) were observed during the one-year follow-up (Fig. 2).

## 3 Discussion

Leishmaniasis in Iran is mainly caused by three species; *L. major*, *L. tropica* and *L. infantum*. *L. major* and *L. tropica* are associated with cutaneous leishmaniasis (CL) whereas *L. infantum* can cause visceral leishmaniasis (VL) (Cincurá et al., 2017). Mucosal leishmaniasis (ML) is mostly associated with *L. brazilliens* (Cincurá et al., 2017). However, several cases have been reported with other species. *L. tropica* has also been linked to mucosal leishmaniasis by a few case reports. It was first described in Saudi Arabia (Morsy et al., 1995), and two cases of mucosal leishmaniasis by *L. tropica* have been reported in Iran. These cases of mucosal leishmaniasis responded to intravenous amphotericin and resolved completely (Shirian et al., 2013).

Mucosal leishmaniasis in new world is a devastating form of leishmaniasis that commonly affects the nasal and oral cavity and may lead to nasal deformity and even destruction of nasal septum (Frischtak et al., 2018). Moreover, it may extend further and involve epiglottis, vocal cord and even trachea and bronchi leading to respiratory failure and death (Carvalho et al., 2018). It may be preceded by the cutaneous lesions or occur as a primary lesion. The standard treatment for ML is pentavalent antimonials; however, its success rate is limited. Other proposed therapies include amphotericin B, miltefosine (Ventin et al., 2018).

Here we reported a *L. tropica* induced mucocutaneous leishmaniasis, resistant to already known treatments; meglumine antimoniate, amphotericin and miltefosine. Thalidomide as an immunomodulator was added to intramuscular meglumine antimoniate (glucantime) therapy, and resulted in complete remission. No recurrences, was observed during the one-year follow-up.

Although production of proinflammatory cytokines, such as Interferon gamma (IFN-γ) and tumor necrosis factor-α (TNF-α), is important for Leishmania killing, an overproduction of these proinflammatory cytokines as well as a decreased ability of IL-10 and TGF-β to modulate this response may lead to severe tissue damage that have been seen in mucosal leishmaniasis patients compared to patients with classical cutaneous leishmaniasis. These abnormalities may be the basis for the pathological findings and the therapeutic challenge observed in this disease, and accordingly, the rational clinical application of immunomodulatory drugs for ML (Ventin et al., 2018). According to immunomodulatory and anti TNF-α activity, adding pentoxifylline to meglumine antimoniate in the treatment of refractory cases of mucosal leishmaniasis is currently recommended (Lessa et al., 2001). However, in our patient, a combination therapy course with oral pentoxifylline and intramuscular meglumine antimoniate did not provide more efficacy than monotherapy with intramuscular meglumine antimoniate.

Thalidomide, once a known drug, deserted due to a series of adverse effects related to its teratogenicity. However, in recent years it has begun to attract attention especially in the field of dermatology for immunomodulatory, anti-inflammatory, and antiangiogenic properties (Franks et al., 2004).

Levels of TNF- α are increased in serum and cultures from patients with mucosal disease that leads to an intense inflammatory reaction, so thalidomide can be useful as TNF-α inhibitor drug (Lessa et al., 2001). Verbon et al. showed that ingestion of thalidomide did not change the level of TNF-α in healthy people (9).

About the effects of thalidomide on Interferon gamma (IFN-γ), an important cytokine in leishmaniasis pathogenesis, some studies demonstrated that an increase in IFN-γ can be observed in response to Thalidomide (Partida-Sanchez et al., 1998). Another study elicited that a single oral dose of thalidomide in healthy people enhances the ability of peripheral blood mononuclear cells (PBMCs) to secrete IFN-γ (Verbon et al., 2000).

In the case of leishmaniasis, no report exists on the use of thalidomide in humans however, an animal study showed treatment potentials of thalidomide in leishmaniasis. In this study, BALB/c mice were infected with L. major and were later treated with a combination of glucantime and thalidomide. Results showed a superior efficacy of combination therapy to glucantime, thalidomide or carrier alone. They also showed that thalidomide effects were mainly due to up-regulation of IFN-γ and down-regulation of IL-10 (Solgi et al., 2006).

Considering the contradictory roles of thalidomide regarding IFN-γ and TNF-α, the results of related studies, and observations from our study, more research is needed to further understand the role of thalidomide in leishmaniasis and its effects on IFN-γ, TNF-α and immune responses of patients with leishmaniasis.

Despite various applications proposed for thalidomide in dermatology, its use is limited by an infamous profile of side effects mainly its teratogenic effects that led to its withdrawal in 1962 (Franks et al., 2004). However many of these adverse effects can be avoided by adhering to guidelines designed in order to decrease its risks especially about teratogenicity.

To the best of our knowledge, this is the first report of the use of thalidomide in the treatment of leishmaniasis in humans. Although it showed promising results in our patient, further studies are required for establishing its efficacy in treatment of leishmaniasis.

## Conflicts of interest

No Conflicts of interest.

## Funding source

No financial support.
